# Antibody-associated responses are linked to increased susceptibility to *Rhodococcus equi* infection in mice

**DOI:** 10.1128/spectrum.02302-26

**Published:** 2026-09-02

**Authors:** Yasunori Suzuki, Shusei Fujiwara, Makoto Sugiyama, Shinji Takai

**Affiliations:** 1 Laboratory of Veterinary Hygiene, Institute of Agriculture, Tokyo University of Agriculture and Technology 13125, Fuchu, Tokyo, Japan; 2 Laboratory of Veterinary Anatomy, Kitasato University School of Veterinary Medicine 73467, Towada, Aomori, Japan; 3 Kitasato University School of Veterinary Medicine 73467, Towada, Aomori, Japan; Instituto Nacional de Tecnologia Agropecuaria, Buenos Aires, Argentina

**Keywords:** antibody, inactivated vaccine, intracellular infection, mouse model, *Rhodococcus equi*

## Abstract

*Rhodococcus equi* is an intracellular pathogen that causes pyogranulomatous pneumonia in foals through replication within macrophages. Protective immunity is generally attributed to T helper 1 (Th1)-type cellular responses, although the mechanisms underlying effective immunity remain incompletely defined. In this study, we assessed the immunological responses to various *R. equi*-derived antigen preparations in a murine model. Immunization with live *R. equi* protected against bacterial proliferation following challenge, whereas immunization with formalin-killed bacteria increased the bacterial burden. Similarly, immunization with bacterial components or secreted products lacking defined antigen specificity resulted in enhanced susceptibility to infection and increased mortality. Antibody analysis revealed that IgG derived from immunized mice significantly enhanced intracellular *R. equi* proliferation *in vitro*. These findings suggest that antibody-mediated uptake may contribute to intracellular survival and replication of *R. equi*. These observations are consistent with an antibody-dependent enhancement (ADE)-like phenomenon in this experimental model. Conversely, live immunization induced a CD8^+^ T cell-skewed response following infection. Collectively, these results indicate that antibody-associated humoral immunity is associated with disease exacerbation, whereas Th1-type cellular immunity correlates with protective immunity against *R. equi* infection.

**IMPORTANCE:**

*Rhodococcus equi* is a major cause of severe pneumonia in foals, and effective vaccines remain unavailable. Recent studies have suggested that antibody responses can have complex effects during bacterial infections; however, their roles in *R. equi* infection remain poorly understood. In this study, we demonstrated that immunization with nonviable *R. equi* antigens induces antibody responses associated with increased bacterial burden and disease severity. These antibodies increased intracellular bacterial loads of *R. equi in vitro*, suggesting that antibody-mediated uptake may facilitate intracellular survival and replication in this experimental model. Contrarily, protective immunity induced by live bacteria was associated with T helper 1 (Th1)-biased responses and early CD8^+^ T cell expansion. These findings highlight the importance of considering the balance between humoral and cellular immunity in vaccine development and underscore the need for strategies that elicit protective Th1-driven cellular immunity against *R. equi* infection.

## INTRODUCTION

*Rhodococcus equi* is a facultative intracellular pathogen and a major cause of pyogranulomatous pneumonia in foals aged 1–5 months (1). The course of treatment is prolonged, and delayed diagnosis is associated with poor prognosis and extremely high mortality rates (2). Moreover, the frequency of *R. equi* infection has been increasing in Japan in recent years, and the disease continues to cause substantial economic losses (2). Furthermore, the recent isolation of this bacterium from suppurative necrotic lesions in pigs, cattle, goats, and humans highlights its increasing significance as a zoonotic pathogen in both public and animal health (3). The pathogenicity of *R. equi* is influenced by its ability to proliferate within macrophages, and virulence-associated proteins (Vaps) encoded on virulence plasmids are involved in proliferation within macrophage phagosomes (4). Vaps promote proliferation by creating a pH-neutral niche within macrophage phagosomes, thereby preventing bacterial killing within the phagosomes (5).

Despite decades of research, effective vaccines for *R. equi* infection remain unavailable. Whereas cellular immunity is thought to be critical for clearance, many aspects of host defense are still poorly understood, and no preventive measures have been established (1). Currently, the only preventive method approved by the United States Department of Agriculture is hyperimmune plasma administration (6). However, its efficacy varies considerably among individuals, and the underlying mechanism of action remains unclear (1). Furthermore, because hyperimmune plasma does not promote the development of an immune memory response, the possibility of future infection following administration cannot be eliminated (7). Research into vaccine development for *R. equi* has long been conducted. Over the past four decades, a wide range of vaccine strategies, including live attenuated, inactivated, subunit, DNA, and vector-based approaches, have been investigated (7). Live vaccines can elicit robust immune responses, but they can also induce clinical symptoms and facilitate environmental shedding of the pathogen, particularly given that *R. equi* is a soil-borne microbe capable of colonizing the rearing environment (8, 9). Although inactivated vaccines are generally considered safe, they often induce weak protective immunity, and in many cases, they fail to promote bacterial clearance following infectious challenge (9–12). Whereas attenuated strains targeting Vap genes or bacterial metabolic pathways have displayed some efficacy in mouse models, none has yet demonstrated sufficient safety and efficacy to support clinical application in foals (7). It is widely believed that protective immunity against intracellular pathogens relies on T helper 1 (Th1)-mediated cellular responses (13). Foals that recover from natural infection typically exhibit Th1-dominant immune profiles, characterized by interferon-γ production and an IgG2a-biased response. Contrarily, severe cases have been linked to Th2-skewed immune responses (14–16).

Mouse models are commonly employed in preclinical studies to assess the immune responses elicited by vaccine candidates. Recently, we established an autologous bioluminescent *R. equi* strain enabling noninvasive longitudinal quantification of bacterial proliferation *in vivo* (17). In this study, we employed this system to analyze immune responses elicited by conventional immunization strategies, particularly examining whether such responses can exacerbate intracellular infection rather than confer protection. Our findings offer insights into how different immune polarization states influence infection outcomes, particularly the exacerbation of intracellular proliferation under Th2-dominant conditions, and provide guidance for the development of effective vaccines for foals.

## RESULTS

### Comparison of *in vivo* growth following immunization with live or inactivated *R. equi*

**Fig 1:**
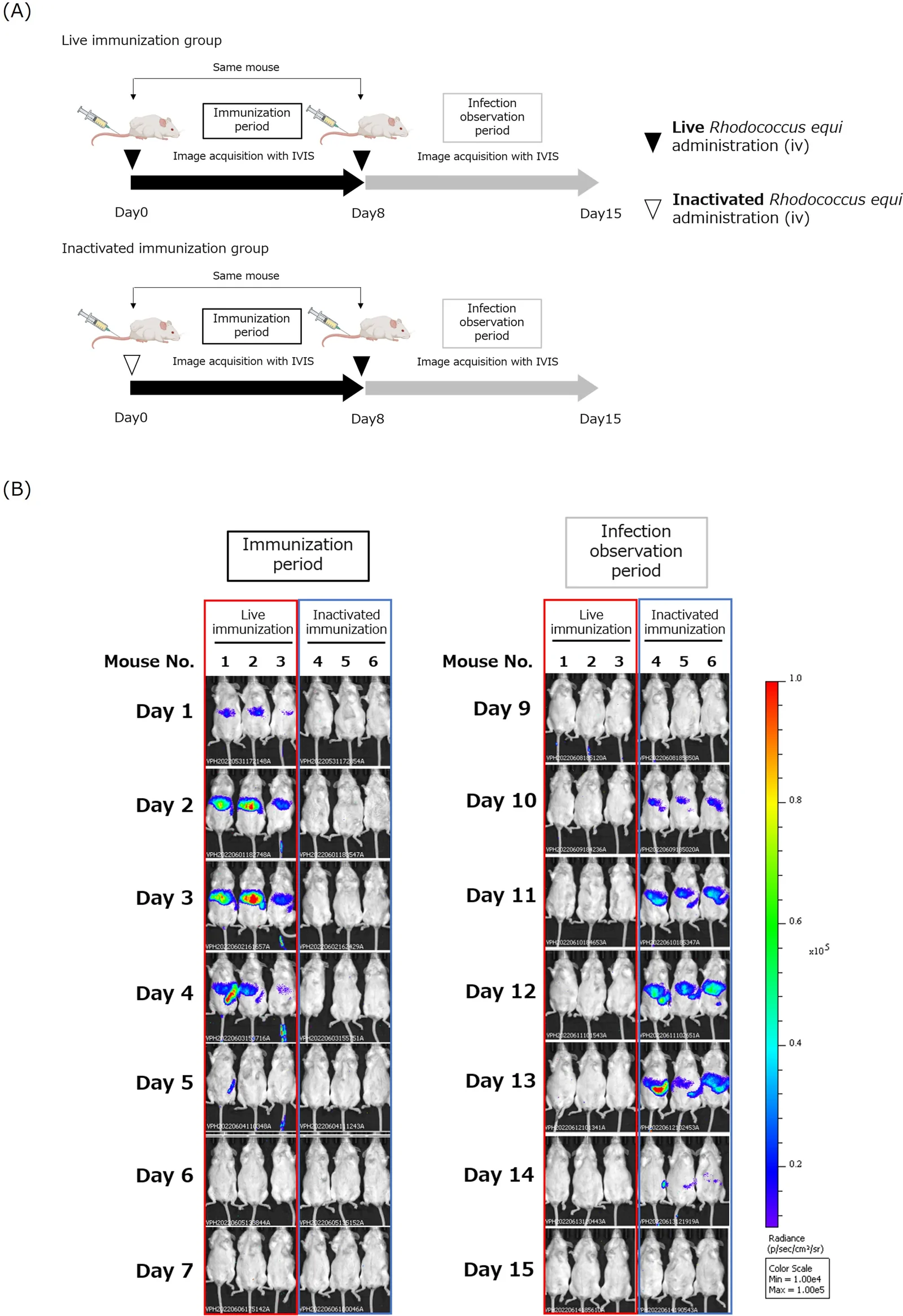
Differences in the *in vivo* growth of *R. equi* in mice immunized with live or formalin-killed bacteria. (**A**) Experimental design. The bacterial strain used was YSNR1 (ATCC 33701 transformed with pYSN1). The inoculation doses were as follows: live bacterium immunization, 1.0 × 10^7^ CFU/mouse; inactivated bacterium immunization, bacteria treated with formalin overnight at a dose equivalent to 1.0 × 10^8^ CFU/mouse. (**B**) Comparison of bioluminescent signals in mice measured using an *in vivo* imaging system. All images were acquired with an exposure time of 5 min. The color scale was standardized with a minimum value of 1.00 × 10^4^ and a maximum value of 1.00 × 10^5^.

Antigen administration and bacterial infection using the YSNR1 strain were performed as outlined in Fig. 1A. Mice immunized with live YSNR1 (1 × 10^7^ CFU/mouse) exhibited transient bacterial proliferation in the liver and spleen, which peaked on day 3 post-immunization and declined to undetectable levels by day 6 (designated as the immunization period in Fig. 1). When challenged with the same live bacteria on day 8, no bacterial growth was observed, indicating the establishment of complete protective immunity (the infection observation period in Fig. 1). Conversely, mice immunized with formalin-inactivated YSNR1 displayed substantial bacterial proliferation in both the liver and spleen during the infection observation period, indicating limited protection (Fig. 1). These findings suggest that live *R. equi* elicits robust protective immunity capable of rapid bacterial clearance, whereas inactivated bacteria fail to induce comparable protective responses.

### Comparison of *in vivo* growth following immunization with culture supernatant proteins

**Fig 2:**
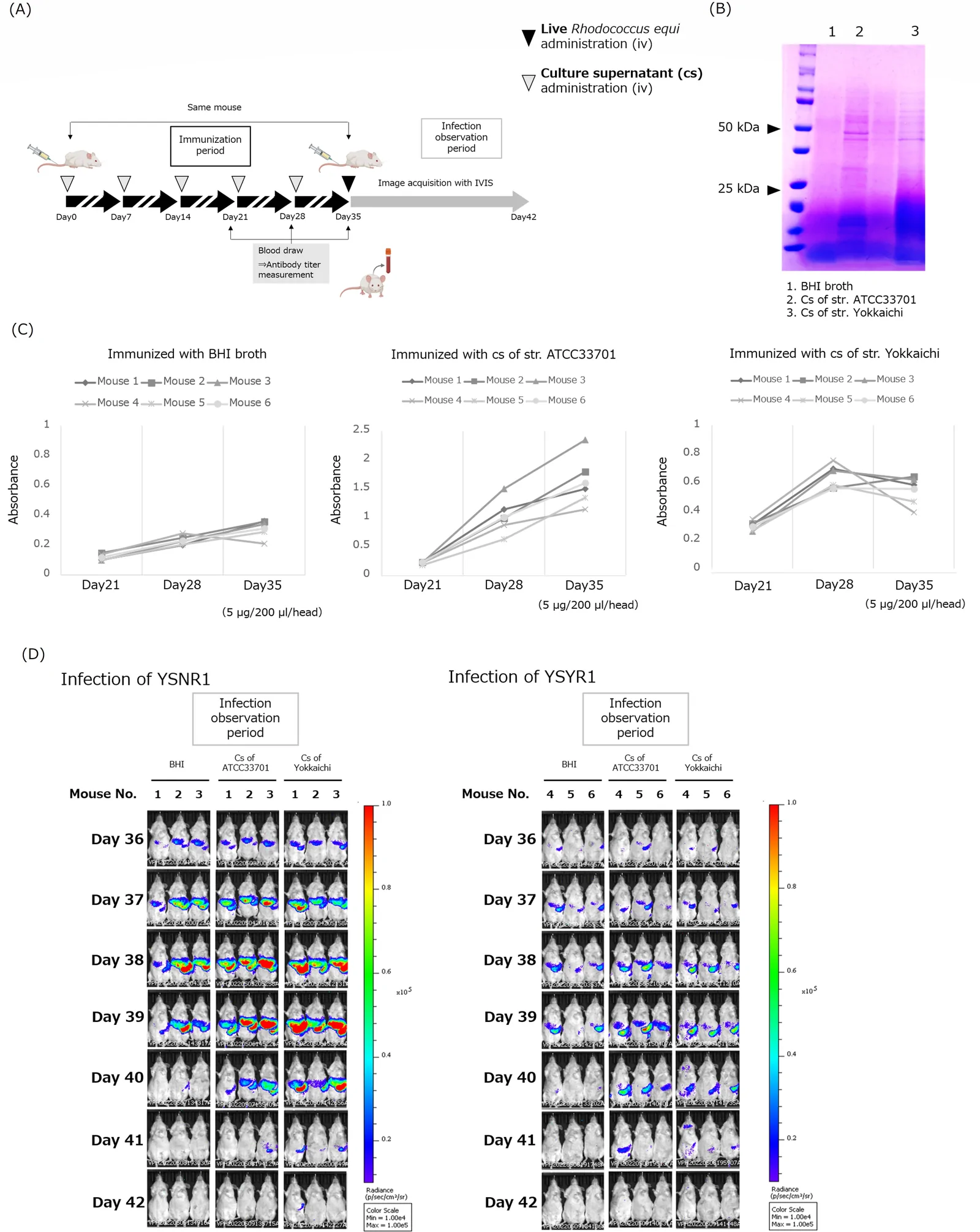
*In vivo* growth differences of *R. equi* in mice immunized with culture supernatant proteins. (**A**) Experimental design. Culture supernatants were prepared from the ATCC 33701 and Yokkaichi strains. The administered dose of the culture supernatant antigen was 5 μg/200 μL per mouse. The inoculum doses of live bacteria were 1.0 × 10^7^ CFU/mouse (YSNR1 strain) and 1.0 × 10^8^ CFU/mouse (YSYR1 strain). (**B**) Sodium dodecyl sulfate-polyacrylamide gel electrophoresis (SDS-PAGE) of concentrated culture supernatant proteins. The purification method is described in the text. (**C**) Antibody titers against each culture supernatant protein. Blood samples were collected the day after antigen administrations 3–5, and antibody titers were measured by ELISA. Further details are provided in the text. (**D**) Comparison of bioluminescent signals in mice. All images were acquired with an exposure time of 5 min. The color scale was standardized with a minimum value of 1.00 × 10^4^ and a maximum value of 1.00 × 10^5^.

Antigen administration and bacterial infection were performed as outlined in Fig. 2A. Mice were immunized with proteins purified from the culture supernatants of the *R. equi* ATCC 33701 or Yokkaichi strain (Fig. 2B). Repeated immunization led to elevated antibody titers against both strains, with the optical density at 492 nm (OD492) ranging from 1.1 to 2.3 for ATCC 33701 and from 0.4 to 0.6 for the Yokkaichi strain (Fig. 2C). Comparison of the mean antibody titers at each immunization point revealed a significant increase in antibody titers with successive immunizations (Fig. S1). Following challenge with live bacteria (YSNR1, a derivative of ATCC 33701, or YSYR1, a derivative of Yokkaichi), mice immunized with both supernatants exhibited more pronounced bacterial proliferation in the liver and spleen and a longer duration of proliferation than vehicle control mice (Fig. 2D). These results indicate that the nonspecific secreted antigens induced an antibody response but failed to confer protective immunity and may have contributed to bacterial persistence.

### *In vivo* growth following immunization with size-fractionated bacterial proteins and *in vitro* effects of IgG from post-infected mice

**Fig 3:**
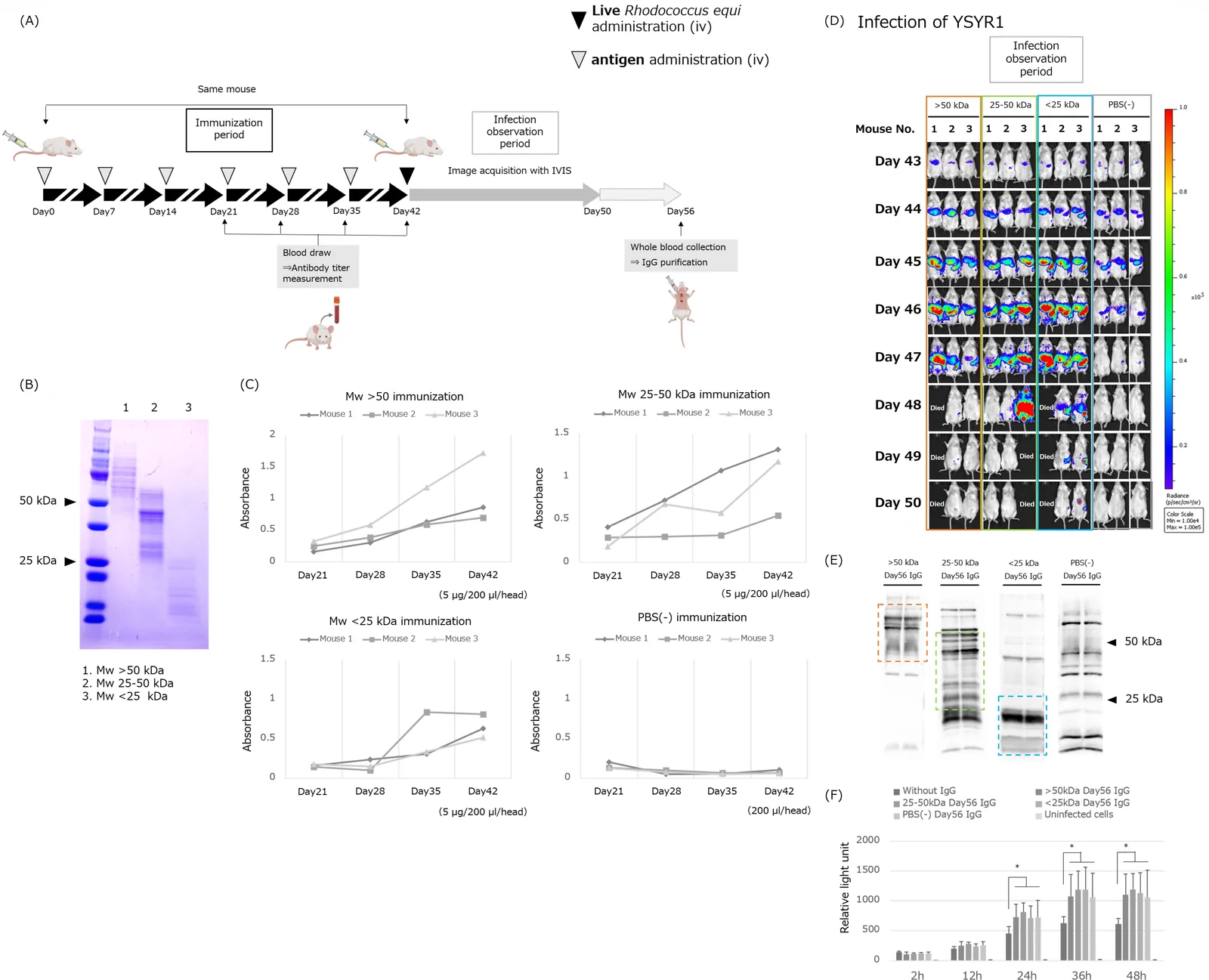
*In vivo* growth differences of *R. equi* in mice immunized with size-fractionated bacterial antigens and the effects of IgG on the intracellular growth of *R. equi* in cultured macrophages. (**A**) Experimental design. The bacterial strain used was YSYR1. Each size-fractionated antigen preparation was administered at 5 μg/200 μL per mouse. The inoculum dose of live bacteria was 1.0 × 10^8^ CFU/mouse. (**B**) SDS-PAGE of administered antigen preparations. The purification methods for each molecular weight fraction of bacterial antigens are described in the text. (**C**) Antibody responses to each size-fractionated antigen. Blood samples were collected the day after antigen administrations 3–6, and antibody titers were measured by ELISA. Further details are provided in the text. (**D**) Comparison of bioluminescent signals in mice. All images were acquired with an exposure time of 5 min. The color scale was standardized with a minimum value of 1.00 × 10^4^ and a maximum value of 1.00 × 10^5^. (**E**) Western blotting using anti-bacterial antigen IgG. SDS-PAGE–separated bacterial antigens from YSYR1 were used as the target. Serum IgG was collected from day 56 mice (post-immunization and post-infection, as presented in Fig. 3A), purified, and used as the primary antibody. (**F**) Evaluation of the intracellular growth of *R. equi* in cultured macrophages in the presence of anti-bacterial antigen IgG. The purified anti-bacterial antigen IgG was added at 150 ng/well during both infection and maintenance culture. “Without IgG” indicates the condition in which macrophages were infected with bacteria and intracellular proliferation was assessed by luminescence measurement without the addition of any IgG samples. Data labeled with asterisks indicate significant differences (*P* < 0.05).

Mice were immunized with size-fractionated bacterial protein preparations derived from lysates of the Yokkaichi strain, consisting of fractions of <25, 25–50, and >50 kDa (Fig. 3A and B). Repeated immunization led to increased antibody titers against each size fraction, with OD492 reaching 0.5–0.7 after six doses (Fig. 3C). Following challenge with YSYR1, all size fraction-immunized groups exhibited substantial bacterial growth in the liver and spleen and stronger luminescence signals than the PBS (−) control group, with one mouse dying in each group (Fig. 3D). These results suggest that nonspecific immunization with bacterial proteins can potentially exacerbate infection. IgG was purified from the sera of mice immunized with each protein fraction and added to J774A.1 macrophage cultures infected with YSNR1. Western blotting confirmed antigen-specific IgG binding to their respective protein sizes (Fig. 3E). All IgG-treated cultures displayed greater intracellular bacterial proliferation than untreated controls, with no significant differences observed among IgG sources (Fig. 3F). These findings support *in vivo* observations that antibodies can promote bacterial proliferation within host cells and further reinforce the concept of antibody-dependent enhancement (ADE), whereby a nonprotective humoral immune response facilitates *R. equi* infection.

### Histological and lymphocyte composition changes following immunization with live and inactivated *R. equi*

**Fig 4:**
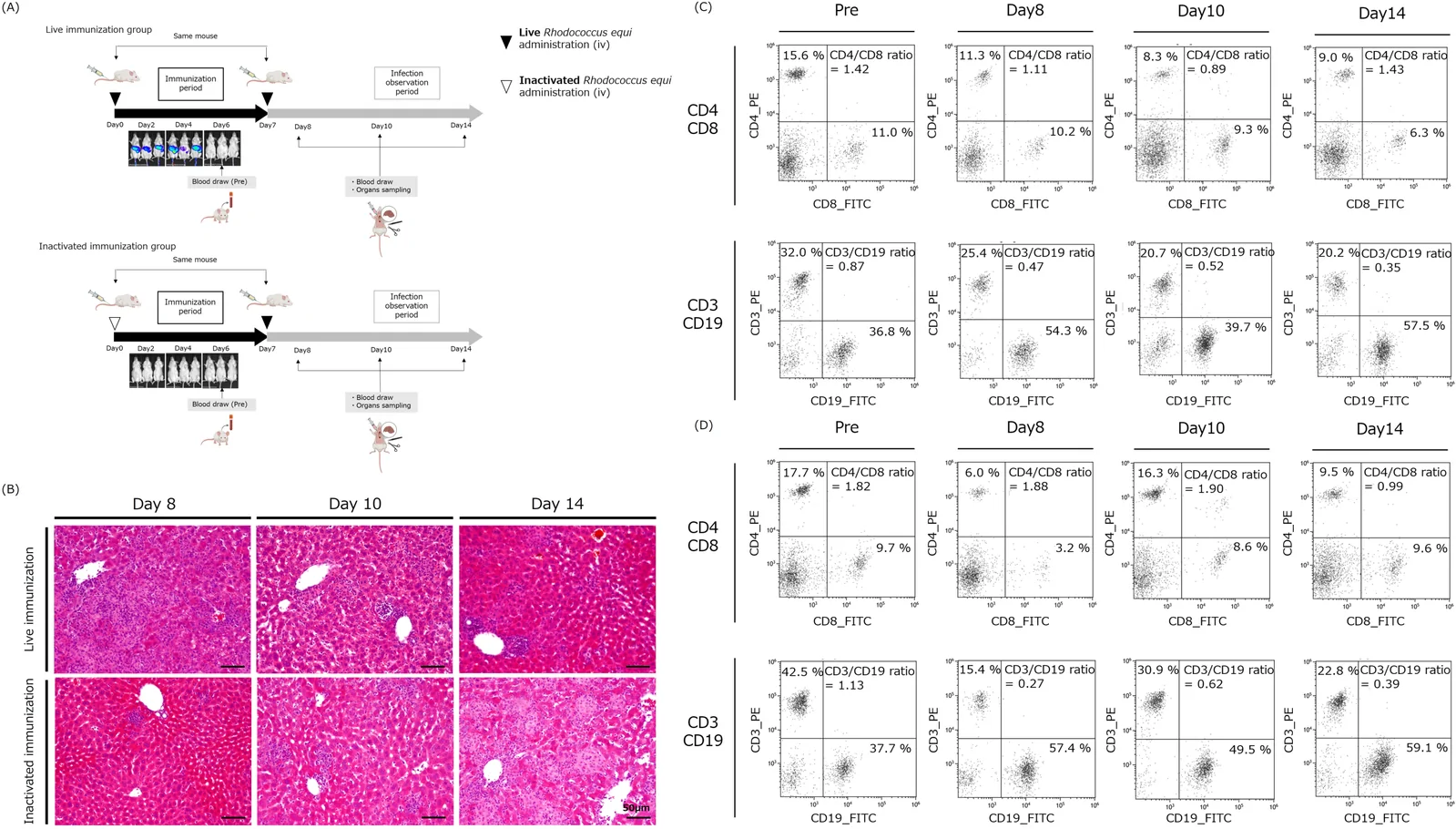
Changes in lymphocyte populations in blood following immunization with live or formalin-killed bacteria. (**A**) Experimental design. The inoculation doses and strains were as follows: live immunization, YSNR1 at 1.0 × 10^7^ CFU/mouse; formalin-killed immunization, YSNR1 treated overnight with formalin at a dose equivalent to 1.0 × 10^8^ CFU/mouse; and live infection. *In vivo* imaging system analysis confirmed bacterial proliferation up to day 4 of the immunization period, followed by a decline in the bacterial burden. (**B**) Hematoxylin and eosin-stained liver sections after *R. equi* infection in mice immunized with live or inactivated bacteria. Bar = 50 µm. (**C and D**) Flow cytometry of peripheral blood from mice immunized with live (**C**) or inactivated bacteria (**D**). Values within the gates (%) represent the mean of biological replicates. Sample sizes were as follows: pre-infection blood samples, *n* = 6 for the immunized groups; and blood samples collected on days 8, 10, and 14, *n* = 2. Statistical analysis was not performed for samples due to the limited sample size.

Histological and flow cytometric analyses were conducted to examine immune cell responses under conditions with and without infection-preventive effects (Fig. 4A). In the live immunization group, inflammatory cell infiltration was observed early, appearing as soon as 1 day post-infection (day 8 in Fig. 4B). Furthermore, signs of tissue recovery were evident 3 days post-infection (days 10 and 14 in Fig. 4B). By contrast, in the group immunized with inactivated bacteria, inflammatory cell infiltration progressively increased following infection, accompanied by an expansion of necrotic foci (Fig. 4B). Peripheral blood was analyzed after live or inactivated bacterium immunization and subsequent infection. In the live immunization group, a decrease in the CD4/CD8 ratio in peripheral blood was observed immediately after infection, whereas in the inactivated immunization group, this change was not detected until 7 days post-infection (Fig. 4B and C). Meanwhile, the CD3/CD19 ratio decreased immediately after infection in the inactivated immunization group (Fig. 4B and C). These findings indicate that early mobilization of CD8-positive cells following live bacterium immunization facilitates bacterial clearance, whereas the induction of B cells (CD19-positive) and subsequent antibody production might have promoted enhanced infection following immunization with inactivated bacteria.

### Analysis of IgG subclasses at the end of the infectious period under different conditions

Concerning IgG subclasses in mice that were protected against infection following immunization with live bacteria, bands corresponding to IgG1, IgG2a, and IgG2b were detected, with the IgG1 band being slightly less intense than the IgG2a band. Contrarily, in mice that did not experience protective effects, IgG1 and IgG2b bands were observed under most conditions, whereas IgG2a bands were generally weaker than the other IgG subclass bands (Fig. S2). These findings suggest that although a Th1 response predominates in the environment favoring bacterial clearance following live bacterium immunization, clearance is less effective under conditions in which a Th2 response predominates.

## DISCUSSION

*R. equi* infection remains a major cause of morbidity in foals on many breeding farms (18). Although foals are frequently exposed to virulent *R. equi* strains on endemic farms, adult horses rarely develop clinical disease, suggesting that protective immunity in adults is mediated by effective adaptive immune responses and immunological memory (19). Vaccination strategies should therefore aim to induce immune phenotypes resembling those of naturally resistant adult horses, underscoring the importance of understanding protective immune mechanisms. In this study, we utilized a bioluminescent *R. equi* ATCC 33701-based strain (YSNR1) to monitor *in vivo* bacterial proliferation and evaluate candidate vaccine molecules. Immunization with live YSNR1 completely prevented bacterial growth following subsequent challenge, confirming the induction of robust protective immunity. Conversely, formalin-killed YSNR1 failed to prevent bacterial proliferation, consistent with previous reports indicating that inactivated vaccines are largely ineffective in foals (9–12). Although live *R. equi* strains harboring the virulence plasmid pVAPA display promise as vaccine candidates, safety concerns, including the risk of clinical disease and environmental shedding, necessitate the development of safer alternatives.

Consistent with the findings after immunization with inactivated bacteria, size-fractionated bacterial proteins and culture supernatant antigens induced strong Th2-biased responses but failed to confer protection (Fig. 2 and 3, and Fig. S2). Notably, purified IgG from mice immunized with size-fractionated proteins enhanced bacterial growth *in vivo* (Fig. 3D) and in macrophages *in vitro* (Fig. 3F), suggesting the possibility of an ADE-like phenomenon. ADE is best known for its role in viral infections, particularly those caused by flaviviruses and influenza viruses (20, 21). In viral infections, ADE occurs when suboptimal antibodies fail to fully neutralize the pathogen, enhancing viral uptake and replication and exacerbating inflammation (22). Moreover, antibody-mediated responses, such as cytokine release and complement activation, although protective, can become dysregulated, driving severe tissue damage and a fatal inflammatory cascade (23). ADE appears to be less common in bacterial infections, and it involves partially distinct mechanisms, including antibody-mediated serum resistance (resistance to complement-mediated lysis), antibody-dependent enhancement of adhesion, antibody-mediated protection from phagocytosis, and virulence enhancement via antibody proteolysis (23). When antibody-opsonized bacteria are more readily internalized by immune cells, such as macrophages, Fc receptor-mediated uptake may be increased. However, some bacteria can survive and replicate within these cells as intracellular pathogens, potentially worsening the infection (24). Wang-Lin et al. (25) reported an ADE-like phenomenon in a mouse pneumonia model of *Acinetobacter baumannii* infection. Using a monoclonal antibody (8E3) specific for the K2 capsular polysaccharide, they observed increased mortality and higher bacterial loads in infected mice. They further demonstrated that shed capsular components bound to 8E3, forming soluble immune complexes that enhanced adhesion to human lung epithelial cells. The authors proposed enhanced bacterial adhesion to and invasion of host cells via interactions between antibody-bacteria complexes and Fc receptors as one possible mechanism underlying this effect. Although the precise mechanism remains to be determined, a similar Fc receptor-mediated process may contribute to the increased bacterial burden observed during *R. equi* infection, particularly given the ability of *R. equi* to survive intracellularly within macrophages.

Flow cytometry demonstrated that live immunization skewed lymphocyte responses following infection toward CD8^+^ T cell dominance, peaking at day 10, along with a Th1-biased IgG response (Fig. 4B). Contrarily, inactivated immunization induced CD8^+^ T cell responses from day 7 onward, accompanied by early B cell expansion and a Th2-biased IgG response (Fig. 4C; Fig. S2). These findings suggest that Th1-type cellular immunity, including CD8^+^ T cell responses, is associated with effective control of *R. equi* infection. This is consistent with protective immune responses reported for infection by *Mycobacterium tuberculosis*, a related actinomycete with similar pathogenic characteristics, in which Th1 cells and CD8^+^ T cells have been implicated as important effector populations (26, 27). Collectively, these results suggest that the selection of antigens capable of preferentially inducing Th1-biased immune responses may be beneficial for developing effective vaccines. The nonclassical cytotoxic T cell responses reported for *R. equi* (28) suggest a potential role for CD1-mediated lipid antigen presentation. Considering the roles of CD1 in lipid antigen presentation and T cell activation (29, 30) and its importance in mycobacterial immunity (31), future studies should examine whether CD1-dependent pathways contribute to immune responses against *R. equi*.

Overall, our findings demonstrate that live *R. equi* immunization conferred protection against bacterial proliferation, whereas inactivated or undefined antigen preparations failed to confer protection and were associated with increased bacterial burden. IgG from immunized mice enhanced intracellular bacterial growth *in vitro* and featured a Th2-associated IgG1-dominant profile, suggesting that an Fc receptor-associated mechanism may contribute to an ADE-like phenomenon. By contrast, protection was associated with a CD8^+^ T cell-biased response. These results suggest that Th1-driven cellular immunity is associated with effective protection against *R. equi*, whereas Th2-biased humoral responses might impair protective immunity.

## MATERIALS AND METHODS

### Bacterial strains

As listed in Table 1, the bacterial strains used in this study included *R. equi* ATCC 33701, originally isolated from a pneumonic foal, and *R. equi* Yokkaichi, originally isolated from cattle with suppurative necrotic lesions (32, 33). In addition, the luciferase-expressing autoluminescent strain YSNR1 (17) and YSYR1, generated by introducing the pYSN1 plasmid [pUC57::aac(3)IV-integrase:: P*_aphII_*-luxABCDE] into the Yokkaichi strain, were used as previously described (17).

**TABLE 1:** *Rhodococcus equi* strains used in this study

Strain names	Description	Reference
ATCC33701	A strain harboring virulence plasmid, pVAPA, isolated from a foal with pyogenic pneumonia	(33)
Yokkaichi	A strain harboring virulence plasmid, pVAPN, isolated from cattle with suppurative necrotic lesions of the liver	(32)
YSNR1	ATCC 33701 transformed with pYSN1	(17)
YSYR1	Yokkaichi transformed with pYSN1	This study

### *In vivo* bioluminescent imaging

Slc:ddY mice were purchased from Japan SLC, Inc. (Shizuoka, Japan). They were housed at 22°C–25°C under a 12 h/12 h light/dark cycle. The YSNR1 and YSYR1 strains were cultured for 48 h at 30°C and adjusted to a density of 1.0 × 10^7^ CFU per animal for YSNR1 or 1.0 × 10^8^ CFU per animal for YSYR1. The bacteria were administered at these inoculation doses. The cultured bacterial strains were administered to each mouse (three animals per condition) via the tail vein (33). The infected mice were housed, and luminescence signals emanating from each transformant were imaged at 1 day intervals using an IVIS200 imaging system (Xenogen/PerkinElmer, Waltham, MA, USA) with an exposure time of 5 min. The total photons emitted were acquired using the Living Image software package. Mice were anesthetized in chambers containing 1.5% isoflurane inhalant (Pfizer, New York, NY, USA).

### Preparation of each antigen

#### Live- and inactivated bacterium immunization

YSNR1 was inoculated into 100 mL of BHI medium (Becton, Dickinson and Company, Franklin Lakes, NJ, USA) and cultured with shaking at 30°C for 48 h. Afterward, 1 mL aliquots were transferred into tubes and stored at −30°C. One vial of the frozen bacterial suspension was thawed, and a 10-fold serial dilution was prepared using sterile PBS (−). Then, 100 μL of each dilution was plated onto standard agar plates and incubated at 30°C for 48 h. Colonies were counted to determine the bacterial concentration per 1 mL of frozen suspension. For inactivated bacterium immunization, the frozen YSNR1 suspension was thawed, and the cells were collected by centrifugation at 5,000 × *g* for 10 min. The supernatant was discarded, and the bacterial pellet was resuspended in 1 mL of 10% formalin solution (FUJIFILM Wako Pure Chemical Corp., Osaka, Japan) and incubated overnight at 4°C. The bacterial cells were then pelleted again by centrifugation at 5,000 × *g* for 10 min and washed twice with PBS (−). The suspension was adjusted to the desired bacterium count for immunization: 1.0 × 10^7^ CFU per animal for live bacteria and 1.0 × 10^8^ CFU per animal for inactivated bacteria.

#### Culture supernatant immunization

The ATCC 33701 or Yokkaichi strain was inoculated into 50 mL of BHI medium and cultured with shaking at 30°C for 48 h. The bacterial suspensions were centrifuged at 4,255 × *g* for 20 min, and the culture supernatants were collected. The recovered supernatant was centrifuged at 4,255 × *g* for 20 min using an Amicon Ultra-1.5 10K Centrifugal Filter (MilliporeSigma, Burlington, MA, USA) to concentrate the culture supernatant protein. A fourfold volume of acetone (FUJIFILM Wako Pure Chemical Corp.) was added, and the mixture was incubated overnight at −20°C. The mixture was centrifuged at 10,000 × *g* for 15 min. The supernatant was removed, and the pellet was resuspended in 500 μL of PBS (−). The presence of the proteins was determined by sodium dodecyl sulfate-polyacrylamide gel electrophoresis (SDS-PAGE) and subsequent Coomassie brilliant blue staining, and the concentration of the purified protein was determined using the Quick Start Protein Assay Kit II (Bio-Rad Laboratories, Hercules, CA, USA).

#### Size-based purification of bacterial antigens and their use in immunization

The Yokkaichi strain was inoculated into 50 mL of conditioned broth (20 g/L peptone, 8 g/L NaCl, and 300 mL/L horse meat extract) adjusted to pH 6.5 and cultured at 37°C for 48 h. The culture was centrifuged at 4,255 × *g* for 20 min, and the supernatant was discarded. The bacterial pellet was washed with 10 mL of cell lysis buffer (40 mM Tris-HCl, 1 M NaCl, 0.1% Triton X-100). Protease inhibitor (100 μL per 10 mL; Nacalai Tesque, Tokyo, Japan) was added, and the suspension was ultrasonicated. The lysate was centrifuged at 15,000 × *g* for 20 min, and the supernatant was collected and concentrated using an Amicon Ultra-15 10K Centrifugal Filter. The concentrated solution was dialyzed against 1 L of dialysis buffer (20 mM HEPES, 100 mM KCl, 0.2 mM EDTA, and 0.5 mM DTT, pH 7.9) with stirring overnight. Proteins were analyzed by SDS-PAGE, followed by Coomassie brilliant blue staining. The stained gel was excised into three size fractions (<25, 25–50, and >50 kDa) and transferred to 1.5 mL tubes. The gel pieces were finely chopped, mixed with 600 μL of extraction buffer (1% SDS, 20 mM Tris-HCl), and incubated with shaking for 16 h. The samples were centrifuged at 3,000 rpm for 10 min, and the supernatants were collected. This extraction step was repeated once. The combined supernatants were concentrated using an Amicon Ultra-15 10K Centrifugal Filter, and a fourfold volume of acetone was added. After thorough mixing, the samples were incubated at −80°C for 2 h, followed by centrifugation at 15,000 rpm for 15 min. The supernatant was discarded, and the pellet was air-dried and resuspended in 100 μL of 0.1% SDS. The recovered protein samples were subjected to SDS-PAGE as previously described to confirm separation into the desired size fractions. The concentration of the purified protein was determined using the Quick Start Protein Assay Kit II.

### Administration of each antigen and measurement of antibody titers in mouse blood using ELISA

The purified protein was diluted in PBS (−) to 5 μg/200 μL per mouse and administered intraperitoneally once weekly. Approximately 30 μL of blood was collected from the tail vein of each mouse on the day following immunization. For ELISA, the purified protein was diluted to 1 μg/mL in PBS (−), and 100 μL was added to each well of a SUMILON MULTI-WELL PLATE (Sumitomo Bakelite, Tokyo, Japan). The plate was incubated at 4°C for 16 h to allow antigen immobilization. Wells were washed three times and blocked with Protein-Free Blocking Buffer (Thermo Fisher Scientific, Waltham, MA, USA) at 300 μL/well for 1 h at 37°C, followed by washing. Serum samples were diluted 1:100 in PBS (−), and 100 μL was added to each well and incubated at 37°C for 1 h. After washing, peroxidase-conjugated goat anti-mouse immunoglobulins (IgG, IgA, and IgM; MP Biomedicals, Irvine, CA, USA) were diluted 1:4,000 in blocking buffer, and 100 μL were added to each well, followed by incubation at 37°C for 30 min. Wells were washed, and 100 μL of OPD substrate solution (FUJIFILM Wako Pure Chemical Corp.) was added, followed by incubation at 37°C for 15 min. The reaction was stopped by adding 100 μL of 3 N sulfuric acid (Kanto Chemical, Tokyo, Japan). Absorbance was measured at 492 nm using an Infinite 200 PRO plate reader (Tecan, Männedorf, Switzerland). Antibody titers were calculated as the difference in absorbance between antigen-coated and uncoated wells.

### Effect of IgG derived from mice after infection on the growth of luciferase-expressing *R. equi* within macrophages

Mice were immunized with purified bacterial antigens selected on the basis of molecular weight, and whole blood was collected via cardiac puncture after the infection period had concluded. Serum IgG was purified using a spin column-based antibody purification kit (Protein G; Cosmo Bio, Tokyo, Japan), and its concentration was determined using the Quick Start Protein Assay Kit II. Antibody titers against bacterial antigens were detected by western blotting. Briefly, the lysate of the Yokkaichi strain was separated via SDS-PAGE and then transferred to polyvinylidene difluoride membranes (GE Healthcare, Chicago, IL, USA) using a HorizeBLOT 4M-R system (ATTO Corporation, Tokyo, Japan). Each collected IgG (×1,000) was used as a primary antibody, and peroxidase-conjugated goat IgG to mouse IgG (whole molecule, ×50,000; MP Biomedicals, Santa Ana, CA, USA) was used as the secondary antibody. Signals were detected using an iBright FL1000 Imaging System (Thermo Fisher Scientific) and ECL Prime Western Blotting Detection Reagent (GE Healthcare). The murine macrophage-like cell line J774A.1 was seeded into white CELLSTAR 96-well plates with transparent bottoms (Greiner Bio-One International GmbH, Kremsmünster, Austria) at 4.0 × 10^4^ cells per well and cultured for 24 h in Dulbecco’s Modified Eagle Medium (DMEM; Nacalai Tesque) supplemented with 10% fetal bovine serum (FBS; Corning, Corning, NY, USA) in a humidified atmosphere containing 5% CO_2_ at 37°C. Cells were then incubated with the YSNR1 strain at a multiplicity of infection of 10 in the presence of purified mouse IgG (150 ng per well), prepared as previously described, for 90 min. After washing with PBS (−), DMEM supplemented with amikacin (40 µg/mL), 10% FBS, and purified IgG (1 µg/mL) was added to each well, followed by further incubation under the same conditions. Intracellular growth was quantified by measuring luminescence using an Infinite F200 PRO system at 2, 12, 24, and 48 h.

### Hematoxylin and eosin staining and flow cytometry

An overview of the blood sampling procedure is presented in Fig. 4A. On the final day of the immunization period (i.e., the day prior to challenge), approximately 200 μL of blood was collected from the tail vein of each mouse as the prechallenge serum. Following YSNR1 infection, whole-blood and spleen samples were collected at 1, 3, and 6 days post-infection. Spleens were fixed in 4% paraformaldehyde (pH 7.4; Sigma-Aldrich, St. Louis, MO, USA) for 17 h, processed using standard procedures, and embedded in paraffin. Sections were stained with hematoxylin and eosin according to previously described methods (34). Blood was added to 1.3 mL of hemolysis buffer (140 mM NH_4_Cl, 10 mM Tris, pH 7.65) and incubated for 5 min at room temperature. After centrifugation at 300 × *g* for 5 min at 4°C, the pellet was washed once with 1 mL of PBS (−) and resuspended in 1 mL of cold PBS (−). Cells were mixed 1:1 with trypan blue and counted. Viable cells (5.0 × 10^5^–1.0 × 10^6^) were resuspended in 100 μL of primary antibody solution for CD4/CD8 or CD3/CD19 double staining using the following antibodies (all from BioLegend, San Diego, CA, USA): PE anti-mouse CD4 (1:50), FITC anti-mouse CD8a (1:100), PE anti-mouse CD3 (1:50), and FITC anti-mouse CD19 (1:100). Samples were incubated on ice for 1 h in the dark, washed once with 1 mL of cold PBS (−), and resuspended in 500 μL of cold PBS (−). Flow cytometry was performed using an FC-500 system (Beckman Coulter, Brea, CA, USA), and the data were analyzed using Kaluza Analysis Software (Beckman Coulter).

### IgG subclass typing

The subclass of purified IgG was determined using a Mouse Monoclonal Antibody Isotyping Kit (Bio-Rad) according to the manufacturer’s instructions. Purified IgG was diluted to 1.0 μg/mL in PBS (−) containing 1% BSA. A 150 μL aliquot of the diluted antibody was added to a tube containing the provided reagent and mixed to dissolve the reagent. The isotype strip was inserted, and the results were read after 5 min. Band intensities were quantified by densitometric analysis using ImageJ software. The values shown represent the relative intensities calculated by dividing the integrated density (IntDen) of each IgG band by the IntDen of the corresponding control line.

### Statistical analysis

Statistical analyses were performed using Statcel4 software (The Publisher OMS Ltd., Saitama, Japan). The luminescence intensity data were initially analyzed by analysis of variance, followed by Dunnett’s post hoc comparisons. *P* < 0.05 was considered significant.

## Data Availability

The data sets used and analyzed during the current study are available from the corresponding author upon reasonable request.

## SUPPLEMENTAL MATERIAL

Supplemental figures spectrum.02302-26-s0001.pdf Fig. S1 and S2.

ASM does not own the copyrights to Supplemental Material that may be linked to, or accessed through, an article. The authors have granted ASM a non-exclusive, world-wide license to publish the Supplemental Material files. Please contact the corresponding author directly for reuse.
