# Supplementary material for: Antibody-associated responses are linked to increased susceptibility to Rhodococcus equi infection in mice
Source: Microbiol Spectr. 2026 Sep 2;14(10):e02302-26. doi: 10.1128/spectrum.02302-26 (PMC13689420; doi:10.1128/spectrum.02302-26)
Supplement: Supplementary file 1 [file spectrum.02302-26-s0001.pdf]

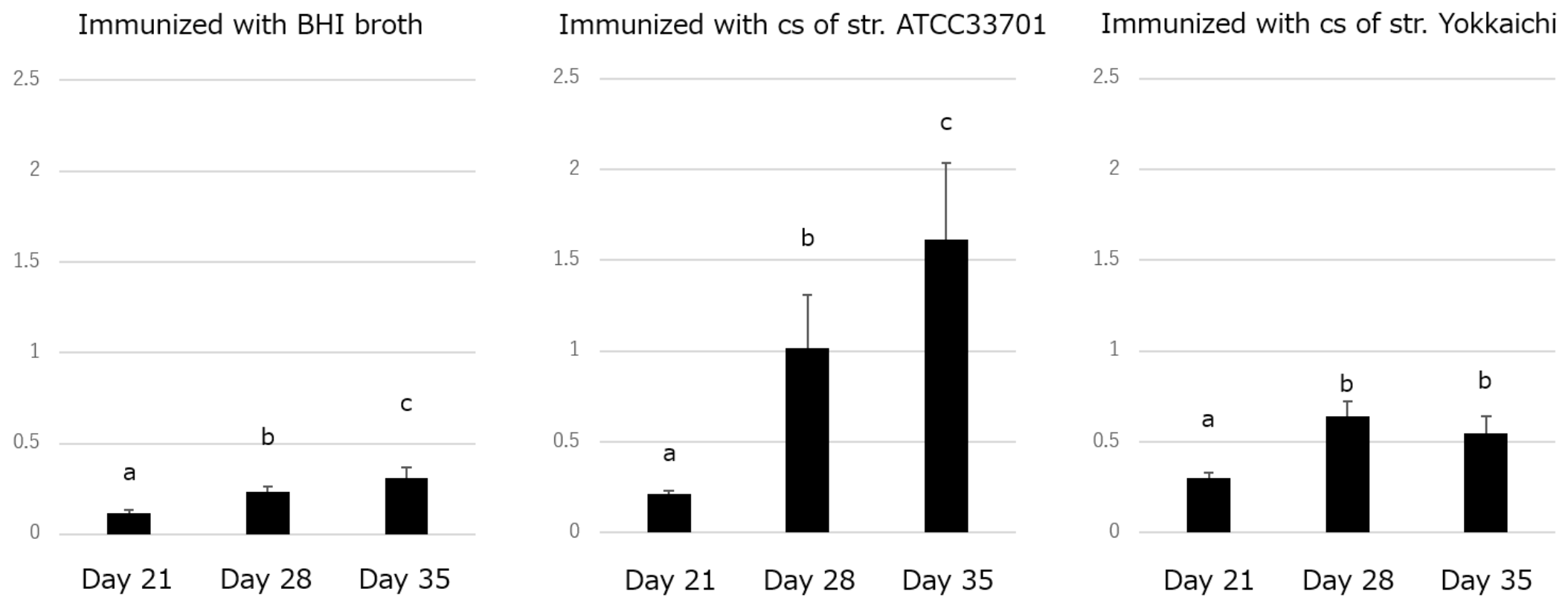

Fig. S1. Statistical analysis of longitudinal changes in antibody titers in mice immunized with culture supernatant proteins.

The antibody titers shown in Fig. 2C were further analyzed to evaluate longitudinal changes in antibody responses. Data are presented as the mean ± SD (n = 6 per group). Statistical analysis was performed using one-way ANOVA followed by Tukey–Kramer multiple comparisons test. Different letters indicate statistically significant differences among groups ($P < 0.05$).

Administration of live or inactivated *R. equi*

	Live		Inactivated	
(Control line)	Detect	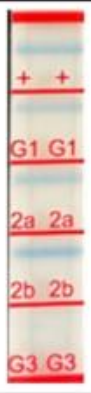	Detect	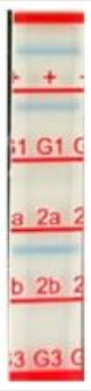
IgG1	0.927		0.975	
IgG2a	0.934		0.751	
IgG2b	1.037		0.961	
IgG3	0.665		0.614	

Administration of culture supernatant from *R. equi*

	Infection of ATCC33701 (collected on Day42)						Infection of Yokkaichi (collected on Day42)					
	BHI		Cs of str. ATCC33701		Cs of str. Yokkaichi		BHI		Cs of str. ATCC33701		Cs of str. Yokkaichi	
(Control line)	Detect	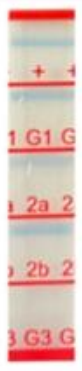	Detect	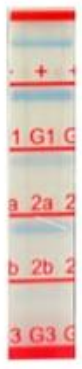	Detect	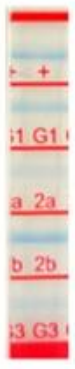	Detect	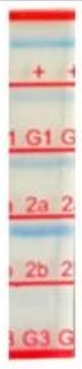	Detect	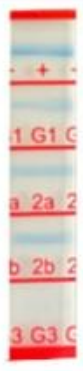	Detect	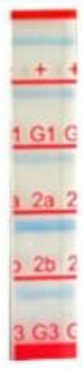
IgG1	1.057		1.124		0.932		0.834		0.816		1.009	
IgG2a	1.021		0.890		0.805		0.916		0.837		0.924	
IgG2b	1.084		0.931		1.066		1.115		0.846		1.208	
IgG3	0.890		0.767		0.843		0.701		0.613		0.904	

Administration of bacterial antigens sorted by protein size

	>50 kDa		25-50 kDa		<25 kDa		PBS(-)	
(Control line)	Detect	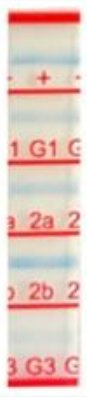	Detect	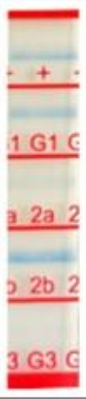	Detect	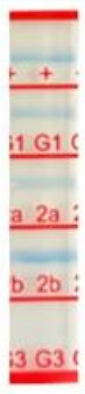	Detect	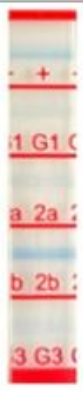
IgG1	0.865		1.011		0.928		0.846	
IgG2a	0.878		0.772		0.869		0.843	
IgG2b	1.027		1.127		1.080		1.128	
IgG3	0.707		0.657		0.642		0.824	

Fig. S2. Serum IgG subclass profiles in mice after immunization and completion of the infection period under each condition.

Band intensities were quantified by densitometric analysis using ImageJ software. The values shown represent the relative intensities calculated by dividing the integrated density (IntDen) of each IgG band by the IntDen of the corresponding control line.
